# Dietary Supplementation with Omega-6 LC-PUFA-Rich Microalgae Regulates Mucosal Immune Response and Promotes Microbial Diversity in the Zebrafish Gut

**DOI:** 10.3390/biology9060119

**Published:** 2020-06-05

**Authors:** Sagar Nayak, Ashraf Al Ashhab, Dina Zilberg, Inna Khozin-Goldberg

**Affiliations:** 1 The French Associates Institute for Agriculture and Biotechnology for Drylands, The Jacob Blaustein Institutes for Desert Research, Ben-Gurion University of the Negev, Midreshet Ben Gurion 84990, Israel; dzilberg@bgu.ac.il (D.Z.); khozin@bgu.ac.il (I.K.-G.); 2 The Dead-Sea and Arava Science Centre, Masada 86910, Israel; ashraf@adssc.org

**Keywords:** arachidonic acid, bacterial community, dihomo-γ-linolenic acid, fish, intestine, immunity, immune function, microbiome

## Abstract

The effect of dietary omega-6 long-chain polyunsaturated fatty acid (LC-PUFA) on host microbiome and gut associated immune function in fish is unexplored. The effect of dietary supplementation with the omega-6 LC-PUFA-rich microalga *Lobosphaera incisa* wild type (WT) and its delta-5 desaturase mutant (MUT), rich in arachidonic-acid and dihomo-gamma-linolenic acid (DGLA), respectively, on intestinal gene expression and microbial diversity was analyzed in zebrafish. For 1 month, fish were fed diets supplemented with broken biomass at 7.5% and 15% (w/w) of the two *L. incisa* strains and a control nonsupplemented commercial diet. Dietary supplementation resulted in elevated expression of genes related to arachidonic acid metabolism-cyclooxygenase 2 (*cox-2*), lipoxygenase 1(*lox-1*), anti-inflammatory cytokine-interleukin 10 (*il-10*), immune defense-lysozyme (*lys*), intestinal alkaline phosphatase (*iap*), complement (*c3b*), and antioxidants-catalase (*cat*), glutathione peroxidase (*gpx*). Microbiome analysis of the gut showed higher diversity indices for microbial communities in fish that were fed the supplemented diets compared to controls. Different treatment groups shared 237 operational taxonomic units (OTUs) that corresponded to the core microbiome, and unique OTUs were evident in different dietary groups. Overall, the zebrafish gut microbiome was dominated by the phylum Fusobacteria and Proteobacteria (averaging 38.4% and 34.6%, respectively), followed by Bacteroidetes (12.9%), Tenericutes, Planctomycetes, and Actinobacteria (at 3.1–1.3%). Significant interaction between some of the immune-related genes and microbial community was demonstrated.

## 1. Introduction

Diet, gut microbiome, and gut-associated immune function are now recognized as key contributors to metabolic processes, pathological conditions, and immune responses affecting the overall health of an organism [1,2,3]. Microbes and microbial products influence both systemic and mucosal immune responses through the interaction of microbe-associated molecular patterns with pattern-recognition receptors in the host. The microbial community in the fish gut contributes to the development, maturation, and modulation of its immune system [4]. Fish microbiota are also known to play key roles in cytokine modulation, B and T leukocyte activation, and immunoglobulin levels [5]. Teleost gut-associated lymphoid tissues have large numbers of antigen-presenting cells that are involved in the initial stages of immune response activation. Diseases can emerge when the gut microbiome is altered by various factors, such as environmental changes, use of antibiotics, and different diets that affect the symbioses and interactions between the gut microbiome and its host. However, the precise mechanisms by which diet in particular can influence host–microbe immune interactions and their impact on fish health remain largely unexplored [6,7]. Zebrafish serve as an excellent model for examining the complex interaction of diet microbiome and the immune function due to a shorter life cycle, high presence of human orthologous genes, and availability of a large array of established genomic tools [8,9].

Long-chain polyunsaturated fatty acids (LC-PUFA) with 20- and 22-carbon chains (C20 and C22, respectively) are important components of human and fish diets. LC-PUFA are divided into omega-3 and omega-6 groups on the basis of the position of the last double bond in the fatty acid carbon chain, serving as precursors for distinct groups of eicosanoids, the lipid mediators of inflammation. Dietary omega-6 fatty acids are generally associated with proinflammatory effects, while omega-3 fatty acids are associated with anti-inflammatory effects. However, recent studies have also demonstrated antioxidant and anti-inflammatory properties of omega-6 fatty acids [10,11,12]. LC-PUFA of both types have been associated with functional roles in ontogenesis, growth, brain development, vision, survival, and disease resistance [13,14]. Omega-6 LC-PUFA include arachidonic acid (ARA, 20:4*n*6) and its precursor dihomo-gamma-linolenic acid (DGLA, 20:3*n*6). Dietary ARA is known to affect lipid metabolism, stress tolerance, disease resistance, and immune function in fish [15,16,17,18,19,20]. Since ARA is a key component of cell membranes, it has important roles in development; growth; and during events of cell damage, injury, and inflammation. However, high concentrations of ARA can be cytotoxic [21] and negatively affect larval performance in certain fish species [22,23]. An optimal amount of ARA is necessary for growth, survival, and immune function, while an overdose may have negative impacts as result of inflammation associated with eicosanoid production. In our previous study, zebrafish fed with diets supplemented with the omega-6 LC-PUFA-rich oleaginous green microalga *Lobosphaera incisa*—either ARA-rich wild-type (WT) or DGLA-rich mutant (MUT) strains—resulted in significant incorporation of the respective fatty acids in the gut [24] and liver [25]. Dietary supplementation also improved immune function and enhanced resistance to *Streptococcus iniae* infection [25]. However, the possible association of this immunomodulatory effect with alterations in the fish gut microbiota is poorly understood.

The dietary omega-3/omega-6 fatty acid ratio plays a pivotal role in human health, and therefore increasing intake of omega-3 fatty acids is recommended by governmental guidelines in many countries [26,27]. The effects of dietary manipulations with omega-3 and omega-6 fatty acids on intestinal health have begun to be revealed. Recent studies in humans and mouse models have highlighted the role of LC-PUFA in forming a synergistic triad with the immune system and the gut microbiome that regulates inflammation and maintains homeostasis [28]. Omega-3 LC-PUFA dietary intake was found to be associated with a higher abundance of genera such as *Bifidobacterium* and *Lactobacillus* [29,30], while another study reported an increase in butyrate-producing genera such as *Blautia*, *Bacteroides*, *Roseburia*, and *Coprococcus* [31]. Elderly mice fed omega-6 diets (supplemented with maize oil rich in the C18 PUFA linolenic acid) exhibited dysbiosis and colitis. However, they were more successful at preventing systemic inflammation than those fed omega-3 diets (supplemented with fish oil [32]). Independent trials showing the effects of omega-6 fatty acids on microbiome structure are relatively scarce compared to those with omega-3, due to the latter’s demonstrated anti-inflammatory properties. The effects of omega-6 LC-PUFA and, in particular, the omega-6 C20 LC-PUFA (ARA and DGLA) on the general microbiome structure and its outcomes have not been previously addressed. Although a few studies have reported the effects of dietary omega-3 LC-PUFA [33,34,35], the impact of the specific omega-6 LC-PUFA ARA and DGLA on the fish microbiome has not been examined.

In our previous study with zebrafish, we demonstrated that ARA- and DGLA-rich diets exert a significant effect on the expression of various immune and inflammatory genes in kidneys, and contribute to better performance of the fish upon bacterial challenge [25]. Since these diets play a critical role in the generation of lipid mediators and signaling compounds, we hypothesized that they can have a localized effect on gut immune function and microbial community. Therefore, the primary objective of this study was to understand whether diets rich in the omega-6 LC-PUFA ARA and DGLA can alter the composition of the gut-autochthonous microbiota and modulate its mucosal immune response. Here, we present the results of next-generation sequencing of 16S rRNA amplicons carried out on gut DNA samples from zebrafish fed diets supplemented with different levels of the ARA-rich microalga *L. incisa* WT and its DGLA-rich MUT strain. The expression of key immune genes, the composition of the microbial community, and their roles in providing protection and improving fish health are discussed.

## 2. Materials and Methods

### 2.1. Experiment Design and Sample Collection

The microalgae *L. incisa* WT and its mutant strain P127 were cultivated in nitrogen-depleted BG-11 medium for 14 days to achieve accumulation of the LC-PUFA ARA and DGLA, respectively, in tricylglycerols. The broken algal biomass was obtained using a method described earlier by Dagar et al. [16]. In brief, algal cell suspension was heated to 80 °C for 1 h under dim light and argon atmosphere to prevent triacylglycerol hydrolysis, cooled to 4 °C, and passed through a grinding bead mill (Dyno-Mill, WAB, Muttenz, Switzerland) followed by freeze-drying to obtain dry powder of broken cells. Experimental feeds were prepared by adding 7.5% and 15% (w/w) dry broken microalgal biomass from *L. incisa* WT and mutant strain P127 (MUT) to a commercial zebrafish diet (Ocean Nutrition, San Diego, CA, USA). A doughy texture was prepared by addition of double-distilled water (DDW) to the mix containing powdered commercial feed and dry broken algal biomass, which was spread evenly on trays and freeze-dried in a lyophilizer, followed by breaking and passing through sieves to obtain the final desired feed size (500 micron). Commercial feed without added microalgae was subjected to a similar procedure and was used as a control feed. The prepared feeds were analyzed for fatty acid composition using a Trace Ultra gas chromatograph (Thermo, Milan, Italy). Total carbon and nitrogen content were determined using an automated elemental analyzer (Thermo). Total carbon and nitrogen content were determined using an automated elemental analyzer (Thermo). The concentration of ARA was 10.8 and 21.8 mg/g feed in the 7.5% and 15% WT-supplemented diets, respectively, and the concentration of DGLA was 6.8 and 13.7 mg/g feed in the 7.5% and 15% MUT-supplemented diets, respectively (Table S1).

Wild-type zebrafish were bred, raised, and maintained at the Fish Health Laboratory at Ben-Gurion University of the Negev, Israel. For the dietary trial adult zebrafish of similar size (6 months old, average weight of 0.27 g) were used. Fish were distributed among 20 glass aquaria (30 fish per tank, 30 L in size) equipped with individual submerged biological filters. Each of the prepared experimental diets was tested in four replicate aquaria. Tanks were siphoned every other day and 10% water exchange was carried out during the entire trial period. Fish were monitored daily and water quality parameters including ammonia, nitrite, and nitrate were measured using colorimetric kits (Merck, Germany). Water quality conditions were maintained as follows: water temperature of 26 ± 1 °C, ammonia (0–0.2 mg L^−1^), nitrite (0–0.25 mg L^−1^), nitrate (0–10 mg L^−1^), and oxygen over 80% saturation. Zebrafish were fed with the different diets at a rate of 2% of body weight per day, in two separate feed applications for a period of 1 month, after which the fish gut was sampled for analyses of gene expression and microbial community. Details of the method of experimental feed preparation, dietary composition analysis, experimental design, and animal husbandry practice are given in our previous study [25]. This experimental trial was approved by the Ben-Gurion University Committee for the Ethical Care and Use of Animals in Experiments (authorization no. IL 58-09-2016).

Fish were starved for 24 h prior to sampling. From each aquarium, 5 replicate fish were sampled for gut-associated gene-expression analysis and an additional 5 fish for gut microbial community analysis, for a total of 20 fish per treatment from 4 replicate aquaria. Samples from replicate aquaria were pooled, 5 fish per pool, i.e., 4 pooled samples per treatment as biological replicates. At sampling, fish were euthanized in clove oil (250 ppm) prior to dissection. The entire zebrafish gut was then carefully removed, avoiding contamination from other smaller organs and visceral fat. Samples collected for gene-expression analysis were immediately placed in RNAlater (500 µL/100 mg fresh weight) at room temperature and left for 6 h at 4 °C before transferring to −80 °C for storage. Gut samples for microbial community analysis were collected in sterile 2 mL round-bottom tubes on dry ice, immediately snap-frozen in liquid nitrogen, and stored at −80 °C until analysis.

### 2.2. RNA Extraction and Gene-Expression Analysis

RNA was extracted from gut samples stored in RNAlater using the SV Total RNA Isolation Kit (Promega, Madison, WI, USA) on the basis of the manufacturer’s instructions. The quality of the total RNA was assessed by gel electrophoresis and it was quantified in a Nanodrop spectrophotometer (Thermo, USA). Reverse transcription was carried out with the Verso cDNA Synthesis Kit (Thermo Scientific, USA) using a mixture of anchored oligo dT (deoxy-thymidine) and random hexamer primers following the manufacturer’s instructions. Quantitative real-time PCR (qRT-PCR) was performed on a Bio-Rad CFX96 Real-Time PCR Detection System with SsoAdvanced Universal SYBR Green Supermix (Bio-Rad, Hercules, CA, USA). Details of primers used in this study are shown in Table S2 (Supplementary Materials section). PCR amplification efficiency E = 10^(−1/slope)^ was determined for individual primer pairs by linear regression analysis of the amount of input cDNA and *C*_t_ values. For this analysis, a mixture of cDNA samples from several samples representing different treatment groups was used in the reaction. E values between 1.9 and 2.1 were obtained for all primers used in the assay. Three technical replicates from each of the four biological replicates were used in the qRT-PCR assay. The reaction mixture consisted of 5 µL SYBR Green Master Mix, 0.5 µL (250 nM) each of forward and reverse gene-specific primers, and 4 µL of diluted template cDNA. The PCR was then performed under the following conditions: initial denaturation at 95 °C for 30 s followed by 40 cycles of 95 °C for 15 s and 60 °C for 15 s. The expression of the target analyzed genes was normalized to the expression of the elongation factor 1α gene (EF1α). EF1α was chosen as the internal control after confirming its stable expression across all dietary treatment groups. Melting-curve analysis was carried out to demonstrate primer specificity and amplification of a single product. Expression values for target genes were normalized to EF1α using the formula 2^(−Δ*C*t)^, where Δ*C*_t_ = average of target gene *C*_t_—average of EF1α Ct.

### 2.3. Extraction of Genomic DNA

DNA was extracted from frozen gut samples using a QIAamp Fast DNA Stool Mini Kit (Qiagen, Germantown, MD, USA) following the manufacturer’s recommendations, with slight modifications to enable initial tissue disruption. Frozen gut tissue was homogenized in a mixer mill (Retsch, Haan, Germany) using glass beads (size 1 mm) and continuous cooling with liquid nitrogen. Following tissue disruption, 1 mL of lysis buffer (InhibitEX) was added to the tubes and from this step onward, samples were processed as instructed in the manufacturer’s protocol. All samples were eluted in 100 µL of ATE buffer (provided with the kit) and later quantified in a Nanodrop spectrophotometer.

### 2.4. Illumina Library Preparation and 16S rRNA Gene Sequence Analysis

Gut samples collected from zebrafish fed four differently supplemented diets (WT 7.5%, WT 15%, MUT 7.5%, MUT 15%) or a control diet (4 replicate samples per diet) were analyzed for microbial community composition; diversity indices and the effect of dietary supplementation were analyzed for 19 collected samples (1 sample of DGLA 15% could not be analyzed due to technical difficulties upon DNA extraction). For microbial community analysis, a two-step PCR library preparation was performed on the extracted DNA for 16S rRNA Illumina sequencing as follows: the first PCR (PCR-I) was performed using the V3–V4 universal 16S rRNA region, in triplicate. Each PCR-I (total 25 µL) contained 12.5 µL KAPA HiFi HotStart ReadyMix (Biosystems, Israel), 0.25 nM of each forward and reverse primer, 10 µL molecular-grade double-distilled water (Sigma, Israel), and 2 µL of (2–100 ng/µL) DNA template. The reaction was performed in an Applied Biosystems thermal cycler (Rhenium, Israel) as follows: initial denaturation at 95 °C for 3 min, and then 36 cycles of 98 °C for 20 s, 55 °C for 15 s, 72 °C for 7 s, and final extension at 72 °C for 1 min. After the PCR-I, gel electrophoresis was applied to verify that all samples were successfully processed. Samples were cut from the gel and cleaned using GenElute Minus EtBr Spin Column following the manufacturer’s protocol (Sigma, Rehovot, Israel). Nucleic acid concentration was then measured for randomly selected samples and recorded. To prepare the library for Illumina sequencing, PCR-II was performed on the PCR-I product. PCR-II was done in triplicate, in a total volume of 25 µL that contained 12.5 µL KAPA HiFi HotStart ReadyMix, 0.75 µL of 300 ng/µL PCR-II primers with Illumina adaptor, 8.75 µL molecular-grade double-distilled water, 2 µL Illumina Miseq barcode, and 1 µL PCR-I product. All PCR-II reactions were performed in an Applied Biosystems thermal cycler: initial denaturation at 95 °C for 3 min, and then 8 cycles of 98 °C for 20 s, 55 °C for 15 s, 72 °C for 7 s, and final extension at 72 °C for 1 min. PCR triplicates were then pooled, and each pooled sample was cleaned using TE SPRI Bead Cleanup (Sigma) following the manufacturer’s protocol, and samples were checked by gel electrophoresis. PCR products were loaded on an Illumina Miseq V3 chemistry lane and sequenced for 300PE read length at the Hebrew University of Jerusalem, Israel.

### 2.5. Sequence Quality Control and 16S rDNA Illumina Sequence Analysis

A series of sequence quality-control steps were performed before data analysis as follows: samples were filtered for PhiX contamination using bowtie2 [36]; incomplete and low-quality sequences were also removed by pairing the two reads using the PEAR program [37]; further quality control, as well as looking for ambiguous bases and mismerged sequences, was carried out using MOTHUR Software V.1.36.1 [38]. Following quality control, sequences were aligned, checked for chimeric sequences, and clustered into different operational taxonomic units (OTUs) on the basis of 97% sequence similarity using “pick_de_novo_otus.py” in Qiime-I software [39]. The sequences were then classified on the basis of Greengenes database V13.8 using the “classify.otu” command; an OTU table was generated using “make_otu_table.py” and sequences were classified as f__mitochondria, c__Chloroplast, k__Archaea; unclassified sequences were removed. All codes used for sequence quality control and classification are listed in the Supplementary Materials section (Files S1–S3).

### 2.6. Statistics and Data Analysis

Results of the qRT-PCR are shown in box plots with values expressed as expression normalized to the internal control gene (Ef1α) ± standard deviation among 4 biological replicates. Relative gene-expression data were statistically analyzed by one-way analysis of variance (ANOVA) using Sigma Plot 13 software (Systat Software Inc., San Jose, CA, USA). Pairwise multiple comparisons were carried out using the Holm–Sidak test (*p* < 0.05 was considered significant). Principal component analysis (PCA) was carried out using the FactoMineR package, and PCA plots were generated using the ggplot 2 package in R studio version 1.14. The OTU table was transformed relative to the total sum of sequences in each sample, and bar plots were plotted for each sample. To check the effect of dietary supplementation on fish gut microbial diversity, we calculated the observed number of OTUs, the Shannon diversity index, and Faith’s phylogenetic diversity for the gut samples. Bar colors were assigned to represent different bacterial phyla, with different shades of the same color representing different OTUs. For significant differences between the different treatments, nonparametric multidimensional scaling (NMDS) plots were constructed on the OTU table by calculating the Bray–Curtis distance matrix, and *p*-values were calculated using the ANOSIM test with 999 permutations. All statistical analyses for microbiome analyses were performed using R V3.4.1 statistical software (RStudio Inc., Boston, MA, USA), and code is available in the Supplementary Materials section (File S1).

## 3. Results

### 3.1. Diet-Induced Changes in Expression of Immune-Related Genes in the Intestine

**Figure 1:**
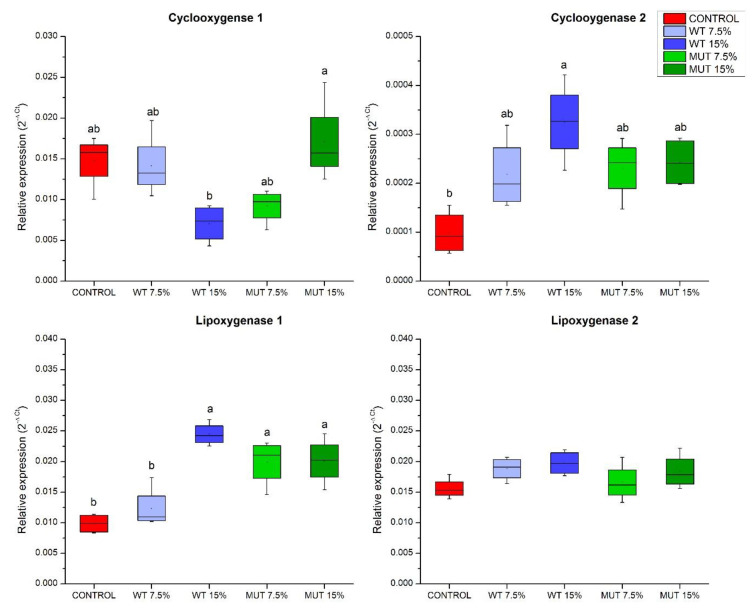
Expression of eicosanoid synthesis genes in the gut of zebrafish fed diets supplemented with *Lobosphaera incisa* wild type (WT) and mutant strain (MUT). Values in box plots show relative gene expression levels normalized to elongation factor 1α (*EF1α*) and expressed as 2^−Δ*C*t^ (*n* = 4 biological replicates, each replicate is a pool of five fish). Different lowercase letters denote significant differences (*p* < 0.05) between treatments.

The expression of two genes involved in the metabolism of ARA and DGLA to their respective prostaglandins (lipid mediators of inflammation and immune modulators was analyzed)—cyclooxygenase 1 (*cox-1*), generally known to be constitutively expressed, and the inducible cyclooxygenase 2 (*cox-2*) (Figure 1). Results showed a significant increase in *cox-2* expression in fish fed the WT 15% diet compared to the control. Expression of *cox-1* did not change significantly between the unsupplemented control and the microalga-supplemented groups. However, in contrast to the general view of constitutive expression of *cox-1* in most tissues, our results showed significantly different levels of expression of this gene in fish that were fed with WT 15% vs. MUT 15% diets. Expression of lipoxygenase (*lox*) genes encoding enzymes involved in the biosynthesis of eicosanoids from LC-PUFA revealed higher expression of lipoxygenase *lox-1* (a homolog of the human *LOX-15*) in all supplemented diet groups except for WT 7.5%, whereas the experimental diets did not seem to affect the expression of *lox-2* (a homolog of the human *LOX-5*).

**Figure 2:**
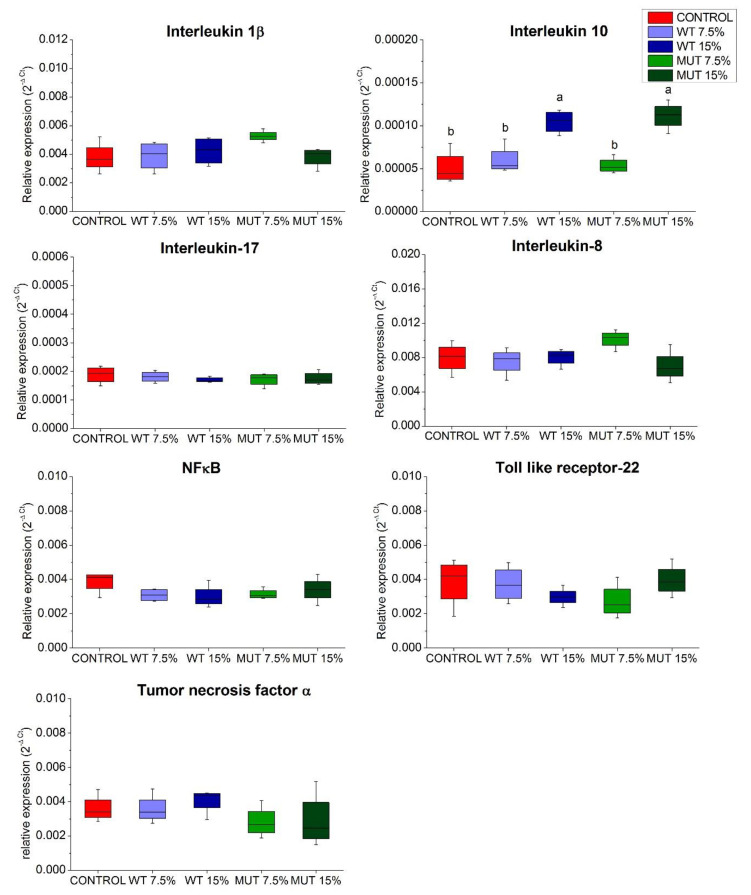
Expression of inflammatory cytokine genes in the gut of zebrafish fed diets supplemented with *Lobosphaera incisa* wild type (WT) and mutant strain (MUT). Values in box plots show relative gene-expression levels normalized to *EF1α* and expressed as 2^−Δ*C*t^ (*n* = 4 biological replicates, each replicate is a pool of five fish). Different lowercase letters denote significant differences (*p* < 0.05) between treatments.

Analysis of genes encoding inflammatory cytokines (Figure 2) revealed that expression of tumor necrosis factor α (*tnf-α*); interleukins 1β (*il-1β*), 17 (*il-17*), and 8 (*il-8*); and the fish family-specific toll-like membrane receptor (*tlr-22*) was either unaltered or at basal levels. Similarly, no effect of dietary microalgal supplementation was observed on the expression of nuclear transcription factor-kappa B (*nf-κB*), which has roles in epithelial homeostasis and inflammation in the gut. However, fish fed the higher levels of supplemented microalgae (WT 15% and MUT 15%) showed significantly higher expression of the gene encoding the anti-inflammatory interleukin 10 (*il-10*).

**Figure 3:**
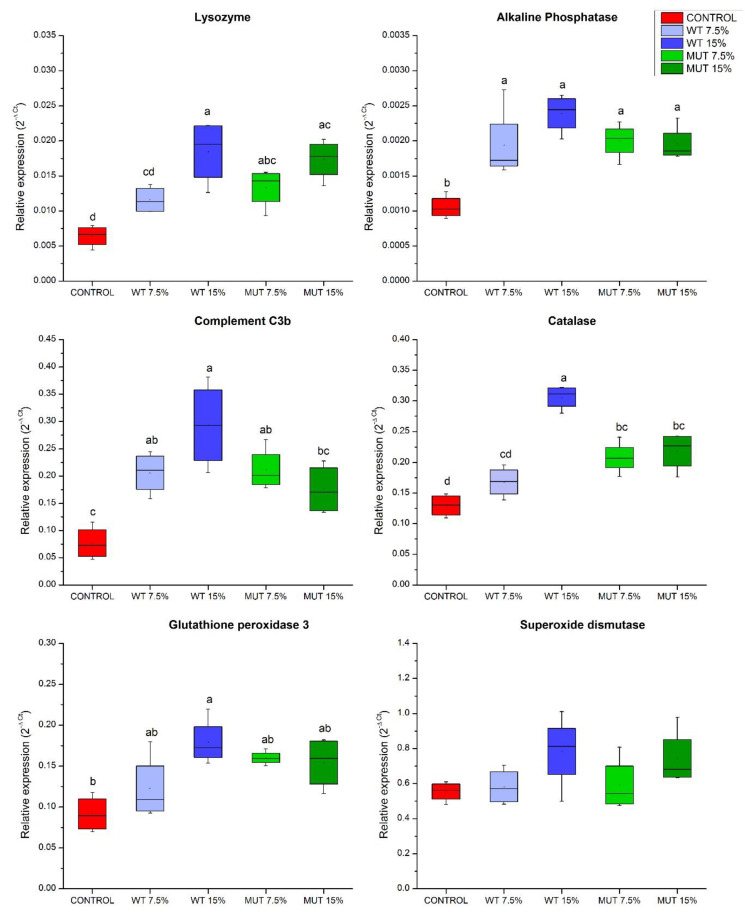
Expression of immune and antioxidant defense genes in the gut of zebrafish fed diets supplemented with *Lobosphaera incisa* wild type (WT) and mutant strain (MUT). Values in box plots show relative gene-expression levels normalized to *EF1α* and expressed as 2^−Δ*C*t^ (*n* = 4 biological replicates, each replicate is a pool of five fish). Different lowercase letters denote significant differences (*p* < 0.05) between treatments.

Results of qRT-PCR showed higher expression of genes related to innate immune function, specifically of the genes encoding lysozyme (*lys*) and the C3b subunit of the complement (*c3b*), in fish fed the microalga-supplemented diets compared to controls (Figure 3). Notably, expression of intestinal alkaline phosphatase (*iap*), which has a crucial role in mucosal defense in the fish gut, was significantly upregulated in fish fed all modified diets compared to the control (Figure 3). Similarly, microalga-supplemented diets increased the expression of the gene encoding catalase (*cat*), an important antioxidant enzyme protecting the cell from oxidative damage caused by reactive oxygen species. A similar increase in expression was observed for the gene encoding glutathione peroxidase 3 (*gpx*-*3*), but this change was only significant relative to controls in fish fed the WT 15% diet. Like catalase, glutathione peroxidase is an antioxidant enzyme that can reduce lipid hydroperoxides and free hydrogen peroxide (Figure 3).

**Figure 4:**
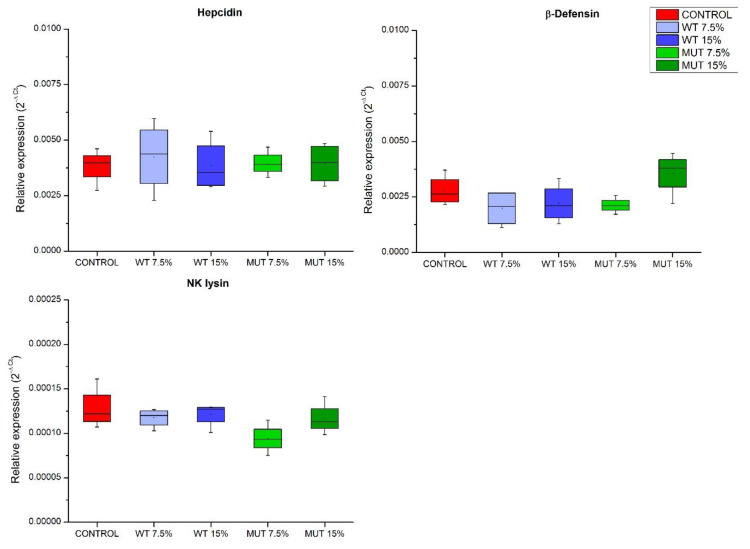
Expression of antimicrobial peptide genes in the gut of zebrafish fed diets supplemented with *Lobosphaera incisa* wild type (WT) and mutant strain (MUT). Values in box plots show relative gene-expression levels normalized to *EF1α* and expressed as 2^−Δ*C*t^ (*n* = 4 biological replicates, each replicate is a pool of five fish). Different lowercase letters denote significant differences (*p* < 0.05) between treatments.

Expressions of genes encoding antimicrobial peptides, important components in innate immunity, were examined (Figure 4). Results indicated no effect of dietary supplementation on the expression of the analyzed antimicrobial peptide-encoding genes, including β-defensin (*def*), natural killer NK cells lysin (*nkly*), and hepcidin (*hep*) genes, as changes were not significantly different compared to controls (Figure 4).

### 3.2. PCA of Gene Expression

**Figure 5:**
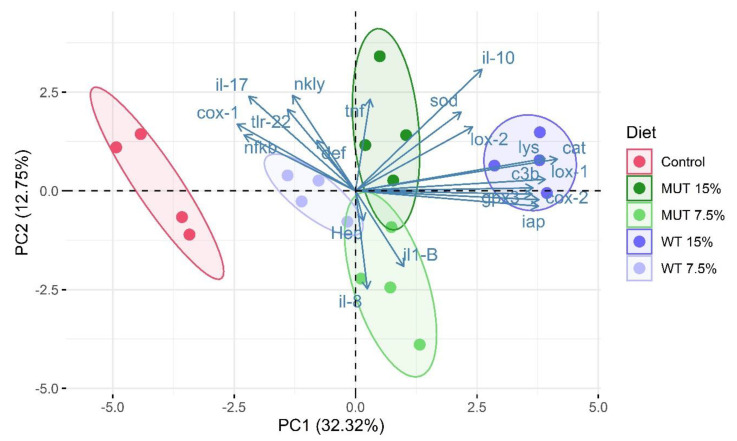
Principal component analysis showing clustering of replicate aquaria (each consisting of a pool of five sampled fish) fed the different diets, on the basis of gene-expression profile. Samples are displayed with respect to the first two components and are colored according to diet. Each colored dot represents an individual biological replicate (*n* = 4). The amount of variance explained by each principal component axis is shown in parentheses. Control, nonsupplemented diet; WT, diet supplemented with wild-type *Lobosphaera incisa*; MUT, diet supplemented with mutant strain of *L. incisa*.

To visualize the gene expression profiles in the gut of zebrafish that are affected by the type of diet consumed (microalga-supplemented vs. nonsupplemented feed), we performed PCA. Biological replicates for each diet clustered closely together on the PCA plot, indicating good consistency between replicates (Figure 5). The first two principal components (PC1 and PC2) explained 45% of the variance. PC1 was characterized by a positive correlation and higher loadings for variables cox-2, lox-1, iap, lys, c3b, cat, gpx, and il-10. Gene-expression patterns of the nonsupplemented (control) groups were clearly discriminated from the microalga-supplemented groups (Figure 5). The separation of treatments based on algal inclusion levels was more prominent in the WT-supplemented groups than in the MUT-supplemented groups.

### 3.3. Microbial Community Changes in Response to Diets

**Figure 6:**
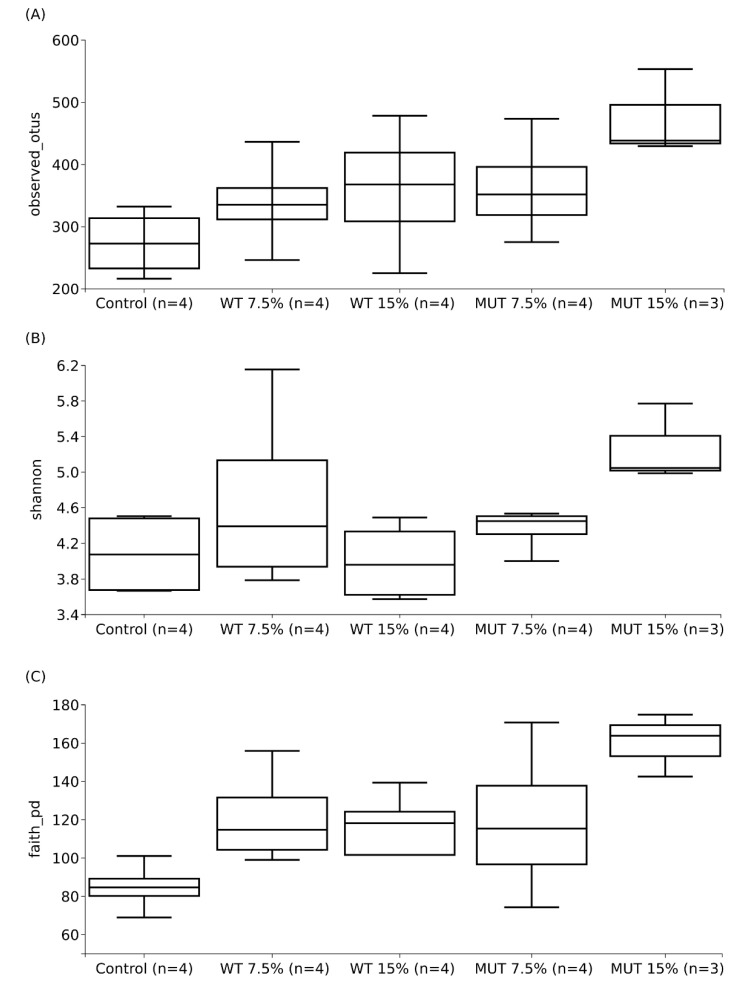
Average microbial diversity indices of (**A**) the observed number of operational taxonomic units (OTUs), (**B**) Shannon diversity, and (**C**) Faith’s phylogenetic diversity, for different diets (control, WT 15%, WT 7.5%, MUT 15%, and MUT 7.5%) and their standard deviations. The number (*n*) on the *x*-axis indicates total experimental replicates; each experimental replicate is a composite of guts from five individual fish. Control, nonsupplemented diet; WT, diet supplemented with wild-type *Lobosphaera incisa*; MUT, diet supplemented with mutant strain of *L. incisa*.

The average number of sequences obtained per sample was 103,964 ± 43,071, while the average retained number per dataset after quality control steps and chimeric-sequence removal was 76,529 ± 37,137. Figure 6 shows the average microbial diversity indices of experimental replicates of each diet (four replicates for Control, WT 15%, WT 7.5%, and MUT 7.5%; three replicates for MUT 15%). The results showed higher diversity indices for microbial communities in fish that were fed the supplemented diets compared to the control feed. In all diversity estimates, the observed number of OTUs, as well as Shannon diversity and Faith’s phylogenetic diversity estimates, showed significantly higher diversity for MUT 15% compared to the control feed (*p* < 0.05). While both the observed number of OTUs and Faith’s phylogenetic diversity showed higher averages compared to the control feed (Figure 6A,C), Shannon diversity did not show clear differences except for the MUT 15%-supplemented diet (Figure 6).

**Figure 7:**
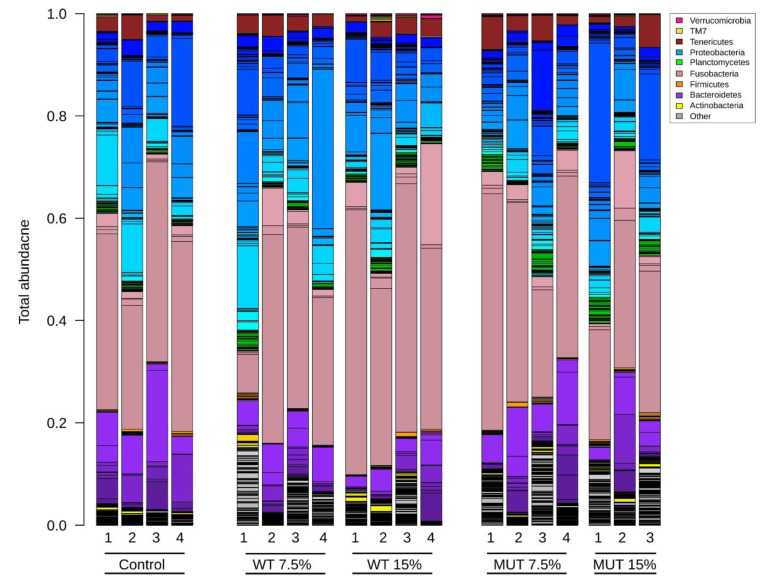
Total microbial community composition in zebrafish fed the different diets. Different colors in each bar represent the different bacterial phylum compositions, while shades of the same color represent different OTUs within the same phyla. Control, nonsupplemented diet; WT, diet supplemented with wild-type *Lobosphaera incisa*; MUT, diet supplemented with mutant strain of *L. incisa*.

**Figure 8:**
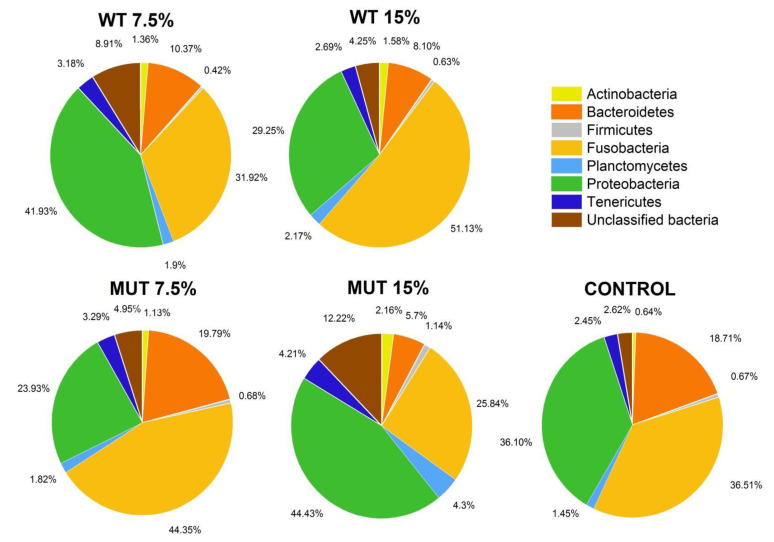
Relative abundance of the major bacterial phyla (>10% of total abundance) in the gut of zebrafish fed the different diets. Values for average relative abundance (%) of the eight most abundant phyla obtained after taxonomic assignment of bacterial 16S rRNA gene sequences. Each phylum is indicated by a different color. Control, nonsupplemented diet; WT, diet supplemented with wild-type *Lobosphaera incisa*; MUT, diet supplemented with mutant strain of *L. incisa*.

The total bacterial community composition in the gut of fish fed the different diets is shown in Figure 7. Five major bacterial phyla were dominant in the zebrafish gut microbiome, starting with the phylum Fusobacteria (38.3% ± 12.5%) which was the most abundant, and followed by Proteobacteria (34.4% ± 11.4%), Bacteroidetes (12.8% ± 8.0%), Tenericutes (3.1% ± 1.7%), Planctomycetes (2.2% ± 1.3%), and Actinobacteria (1.4% ± 1.0%). A fraction of unclassified bacteria (6.2% ± 4.8%) was also observed in the gut microbiome. The relative abundance of the top eight bacterial phyla among the different dietary treatment groups is shown in Figure 8. High variability of relative abundance values for phyla was observed in the microbiota of fish fed both the WT- and MUT-supplemented diets (Table S3). Despite this variability, the WT 15% diet promoted higher abundance of Fusobacteria (*p* < 0.05) than the MUT 15% diet. The relative abundance of Proteobacteria, Bacteroidetes, and Firmicutes, however, did not differ significantly between diets at the end of the feeding period. Remarkably, the MUT 15% diet promoted a higher abundance of the phylum Planctomycetes (*p* < 0.05) than the control diet. Moreover, average relative abundance values for Actinobacteria and unclassified bacteria were higher in fish fed the microalga-supplemented diets compared to the control diet, although statistical significance could not be established due to variance (Table S3, Figure 8).

**Figure 9:**
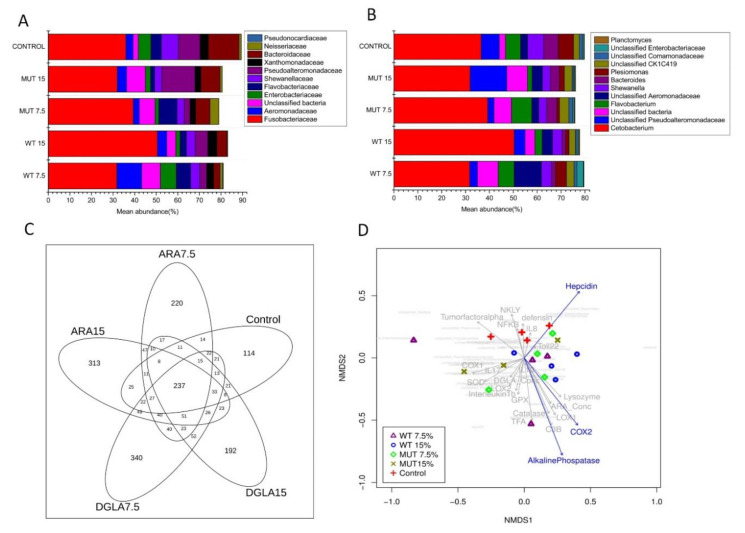
Relative abundance of bacteria at the family (**A**) and genus (**B**) levels in the gut of zebrafish fed different diets. Values for average relative abundance (%) of only the dominant families or genera obtained after taxonomic assignment of bacterial 16S rRNA gene sequences are shown. Individual phyla are indicated by different colors. (**C**) Venn diagram showing unique and shared bacterial OTUs at 97% sequence similarity among different dietary treatment groups. The numbers in the overlapping circles indicate OTUs shared by the experimental groups. (**D**) Nonparametric multidimensional scaling (NMDS) ordination illustrating the effects of different dietary supplementation (WT, MUT at 7.5 and 15%, and control) on fish gut bacterial community. The plot also includes the tested significant and nonsignificant immune gene expression profiles (blue and gray arrows, respectively) and the microbial species distribution (97% of total species abundance, in light gray in the background). Control, nonsupplemented diet; ARA, diet supplemented with wild-type *Lobosphaera incisa*; DGLA, diet supplemented with mutant strain of *L. incisa*.

At the family level, the microbiome was dominated by Fusobacteriaceae, followed by Bacteroidaceae, Pseudoalteromonadaceae, Flavobacteriaceae, Aeromonadaceae, Shewanellaceae, Enterobacteriaceae, and Xanthomonadaceae (Table S3, Figure 9A). The genus *Cetobacterium* dominated the gut microbiota in all diet groups, constituting 36.5%, 31.7%, 50.4%, 39.3%, and 31.9% in the Control, WT 7.5%, WT 15%, MUT 7.5%, and MUT 15% diets, respectively (Table S3, Figure 9B). Other predominant genera of the intestinal microbial community were composed of unclassified Pseudoalteromonadaceae, *Flavobacterium*, unclassified Aeromonadaceae, *Shewanella*, *Bacteroides*, and *Plesiomonas* (Table S3, Figure 9B). Although statistically significant differences were not observed at the genus level, microalga-supplemented diets tended to have decreased abundance of *Bacteroides*, *Plesiomonas*, and *Shewanella* compared to the control diet. The decrease in the abundance of *Bacteroides* was mostly evident when comparing the WT and MUT diets.

A total of 237 OTUs (species/genus level) were shared among fish from different dietary groups, corresponding to the core microbiome in the zebrafish (Figure 9C). Analysis of the core microbiome showed the presence of 220 and 313 unique OTUs in the WT 7.5% and WT 15% groups, respectively, while 340 and 192 unique OTUs were present in the MUT 7.5% and MUT 15% groups, respectively. Interestingly, the microbiome of fish fed the control diet also showed 114 unique OTUs, most of which were of very low abundance (<10%).

To better illustrate the effect of different diets on the microbial communities and to assess whether they differ significantly, we generated an ordination using a NMDS plot based on the Bray–Curtis distance matrix of the microbial profile for each sample (Figure 9D). The NMDS plot showed clear separation of the microbial communities for the control, while the WT 7.5% and 15% diets did not show a clear separation and overlapped with the MUT 7.5% and 15% diets. The NMDS also showed the effect of dietary supplementation on gene-expression profiles and their correlation with microbial community structure. Expression of *tnf-α*, *nkly*, *nf-κB*, *il-8*, and *def* was positively correlated with the control diets, whereas *cox-1*, superoxide dismutase (*sod*), *il-17*, *lox-2*, *il-1β,* and *gpx-3* expression correlated with the MUT diets. However, *cat*, *c3b*, *cox-2*, *lox-1*, *lysozyme*, and *iap* correlated positively with the WT diets. Notably, expression of *iap*, *cox-2*, and *hep* showed a significant effect on gut microbial community structure (*p* = 0.008, 0.047, and 0.05, respectively).

## 4. Discussion

Studies on the effects of microalga-supplemented diets on the host microbiome are very rare. Previous studies analyzing fish microbiome alterations in response to dietary fatty acid supplementation used mainly omega-3 LC-PUFA-producing microorganisms, such as *Schizochytrium* [35], and the microalgae *Nannochloropsis gaditana* [34] and *Phaeodactylum tricornutum* [33]. This is the first report of the effects of omega-6 LC-PUFA-rich *L. incisa* on the fish gut microbiome. Results from gene expression assays and high-throughput 16S rRNA gene sequencing demonstrated that dietary supplementation with *L. incisa* WT and MUT strains plays a crucial role in forming a synergistic triad, together with the gut mucosal immune system and the microbiome. It should be noted that various components in the microalgae, additional to the LC-PUFA-rich oils, might contribute to this effect, including β-carotene, vitamins, and other active pigments [16,40]. The unique composition of omega-6 LC-PUFA in the microalgae in this study, which have been shown to be incorporated into the fish’s organs [24], is of particular interest due to its known substantial effect on fish immune function [25]. Our findings show for the first time that supplementation with omega-6 LC-PUFA can attenuate inflammation and enrich microbiome diversity.

### 4.1. LC-PUFA-Rich Diets Promote COX and LOX Pathways in the Gut

Higher expression of *cox-2* and *lox-1* (homolog of the human *LOX-15*) in the gut suggests enhanced conversion of dietary ARA and DGLA to their respective associated eicosanoids. The downstream products of metabolism from these pathways regulate the dynamics of both inflammation and its resolution. The *cox-2* gene encodes COX 2, a key inducible enzyme catalyzing the conversion of ARA to prostaglandin E2 (PGE2). Higher levels of *cox-2* transcript suggest increased production of eicosanoids such as PGE2, associated with inflammation, as well as resolution of acute inflammation [41]. Studies have shown that endogenous PGE2 can promote mucosal integrity and immune stimulation during inflammation [42]. Studies have also demonstrated that PGE2 production can inhibit 5-LOX, decreasing the proinflammatory 4-series LTs (leukotrienes), and at the same time induce 15-LOX, thus promoting the formation of lipoxins that act to resolve inflammation [43,44]. The results of our study are consistent with these findings, as they showed higher expression of *lox-1* in all groups receiving supplemented diets except for WT 7.5%, with no apparent effect on the expression of *lox-2* (homolog of the human *LOX-5*). We therefore suggest that *L. incisa*-supplemented diets can improve immunocompetence in zebrafish by regulating inflammatory gene expression. The results obtained from this research on zebrafish could also be relevant to other important farmed fish species.

### 4.2. Supplemented Diets Create an Anti-Inflammatory Environment in the Gut

IL-10 is an anti-inflammatory cytokine that is known to suppress inflammatory cytokines [45]. Expression of *il*-*10* with the supplemented diets could be a major factor affecting survival, as it can induce the repair of mucosal damage during inflammation and regulate monocyte/macrophage populations. Interestingly, extracts of sea cucumber (*Apostichopus japonicus*), known for its many therapeutic effects, fed with cultivated omega-3 LC-PUFA-producing species of *Schizochytrium* and the microalga *Nannochloropsis oculata* upregulated IL-10 expression in mouse splenocytes [46]. IL-10 has been identified in a number of teleost fish and its key anti-inflammatory activities—phagocyte stimulation, B-cell differentiation, promotion of memory T cells, and roles in antibody secretion—have been established in the European common carp (*Cyprinus carpio*) [47]. Although the specific components in our microalgal diet responsible for stimulation of the anti-inflammatory IL-10 have yet to be established, they may be related to the rich ARA and DGLA contents in the microalgal biomass. We hypothesize that the increased expression of *il-10* in this study could be due to higher inclusion levels of both WT and MUT supplements, thus resulting in an anti-inflammatory effect in the fish intestine. Evidently, the expression of proinflammatory cytokines, including *tnf-α, il-1β, il-17, il-8*, *tlr-22*, and *nf-κB*, was not affected by the supplemented diets.

### 4.3. Activation of Factors Associated with Antibacterial Effect and Protection from Oxidative Damage in the Gut

The marked increase in the expression of two key genes involved in the innate immune defense—lysozyme and complement—indicated activation of the gut mucosal defense system. Lysozyme and complement are important components of the teleost innate immune system, which acts directly on bacterial pathogens by inducing cell-wall disruption [48,49]. Lysozyme lyses Gram-positive bacteria and can also lyse Gram-negative bacteria by synergistic activity with complement factors [50]. The complement C3 component is a central protein in the complement system that principally promotes phagocytic activity by binding to bacterial liposaccharides through an alternative pathway, or through lectins and surface proteins such as C-reactive protein (CRP) [51]. A similar increase in expression of both lysozyme and complement genes was observed in the kidneys of zebrafish, after feeding with *L. incisa*-supplemented feed [25]. The possible reason for enhanced activity of these proteins in the gut of fish that consumed *L. incisa*-supplemented diets might be the high content of omega-6 LC-PUFA (ARA and DGLA). Similar to lysozyme and complement, higher expression of the IAP-encoding gene indicated stimulation of mucosal defense in the gut, as IAP is known to play a crucial role in resolving inflammation by dephosphorylating proinflammatory molecules, including bacterial lipopolysaccharides [52,53]. Statistical analysis (NMDS) of the microbiome also revealed a significant effect of IAP on microbiome composition between diets. The previously demonstrated correlation between dietary fatty acids and IAP on the one hand, and recognition of IAP as a key modifying factor of the gut microbiome on the other, might explain this relationship [54,55].

The elevated expression of cat and gpx genes in the gut might be due to the high levels of LC-PUFA in the supplemented diets, which are highly susceptible to oxidation. Catalase is a tetrameric porphyrin-containing enzyme that catalyzes the conversion of H_2_O_2_ to water and molecular oxygen, thereby maintaining cellular redox balance; glutathione peroxidase plays an important role in detoxifying lipids and hydroperoxides and protecting cellular components from oxidative damage during events such as phagocytosis or physiological metabolism [56,57]. Alternatively, the increased expression of cat and gpx may indicate higher scavenging potential for reactive oxygen species inside the gut, thereby offering a defense mechanism against oxidative damage. It is important to note that additional components in the microalgae can induce upregulation of the expression of these genes.

### 4.4. Microalgal Diets Modulate Gut Health and Infection Outcome through Enhancement of Microbial Species Diversity

The feeding of supplemented diets increased microbial diversity and thus affected species composition in the zebrafish gut. A similar increase in diversity of gut bacteria has been reported in studies with tilapia (*Oreochromis niloticus*) and rainbow trout (*Oncorhynchus mykiss*) fed a whole-cell dietary *Schizochytrium* meal [35,58]. The increase in diversity in the present study could be an adaption to enable digestion and assimilation of the added microalga-derived ingredients, including cell-wall components, polysaccharides, and lipids with omega-6 LC-PUFA (DGLA or ARA), substrates that may have attracted colonization by relatively less abundant (rare) bacterial taxa, in addition to the previously colonized microbiota. This is supported by our observation of many unique OTUs in fish fed the supplemented diets that were less abundant in the total microbiome. It is possible that the higher number of unique OTUs (bacterial species) and their secreted metabolites in fish fed the supplemented diets affect mucosal function and provide protection against bacterial infection (as demonstrated in our previous study for infection with *Streptococcus inaie* [25]). In general, more diverse communities can exert a protective effect in the host by increasing the probability of having a species with an antagonistic effect toward foreign pathogens and invaders. This is equally supported by the fact that higher gut microbial diversity is considered a biomarker for fish health and metabolic capacity, whereas lower diversity and instability of the microbial population has been associated with disease states in fish [59,60].

### 4.5. Dietary L. incisa Supplementation Has No Deleterious Effect on the Native Microbiome

The occurrence of Cetobacterium as a dominant genus in all samples of zebrafish gut is in agreement with previous studies that reported dominance of this genus in a range of freshwater fish species, including *Oreochromis niloticus*, *Cyprinus carpio*, and *Arapaima gigas* [61,62,63]. Although the exact reason for their dominance in the microbiome is not understood, studies have demonstrated an association between Cetobacterium species and the production of vitamin B12 in a number of freshwater fish species [64]. In addition, bacteria of the genus Bacteroides—another group of dominant taxa in the fish gut—are known for their ability to degrade a wide variety of plant polysaccharides [65] and produce short-chain fatty acids that play an important role in resolving gut inflammation [66]. The higher abundance of Fusobacteria and Proteobacteria in all groups as dominant phyla is in agreement with previous studies that reported their higher abundance in the gut of omnivorous fish as compared to carnivorous and herbivorous fish. These phyla have also been detected at varying levels in studies of intestinal microbiota in zebrafish and other teleost species [63,67,68]. Moreover, these phyla have been identified as potential members of the core zebrafish gut microbiota [68]. Overall results of the microbiome analysis indicated that dietary supplementation with *L. incisa* strains does not disturb the core species and maintains the equilibrium of dominant microbial taxa native to zebrafish, thus providing stability (preventing dysbiosis) and promoting resilience to stress/challenge. Our results are also in agreement with a recent study that showed that consumption of whole-cell *Chlamydomonas reinhardtii* biomass in humans has no adverse effect on microbial composition of the gut and promotes healthy gastrointestinal function [69]. One of the limitations for microbiome analysis in this study was the relatively small sample size per dietary group. A higher number of samples could have increased the statistical power, reduced the variance within treatment groups, and enabled better differentiation of bacterial taxa within the experimental diets.

## 5. Conclusions

Results from this study demonstrate that dietary supplementation with the LC-PUFA-rich microalga *L. incisa* can modulate zebrafish mucosal immune function and increase gut microbial diversity, promoting improved gut health and potentially providing protection from microbial challenge. Results suggest that the DGLA and ARA contents in the microalgae are associated with the modulation in gut immune function and microbiome. On the basis of this and our previous studies on properties attributed to *L. incisa* and its omega-6 LC-PUFA content of ARA and DGLA, dietary supplementation with this microalga holds promise as a potential treatment for the prevention of gut-associated infections and immune-related diseases.

## Supplementary Materials

The following are available online at https://www.mdpi.com/2079-7737/9/6/119/s1, Table S1: Fatty acid composition and content of experimental diets. Table S2: Primers used in qRT-PCR. Table S3: Mean relative abundance of dominant taxa at various taxonomic levels. File S1: Code. File S2: OTU table. File S3: Barcode sequences.
